# Hiding Beaver Tail Liver: A Rare Anatomical Variant of the Liver

**DOI:** 10.7759/cureus.62665

**Published:** 2024-06-19

**Authors:** Vinay Sharma, Padamjeet Panchal

**Affiliations:** 1 Anatomy, Muzaffarnagar Medical College, Muzaffarnagar, IND; 2 Anatomy, All India Institute of Medical Sciences, Patna, Patna, IND

**Keywords:** beaver tail, sliver of liver, anatomic variation, morphology, spleen, liver

## Abstract

Accessory liver lobes are indeed morphological variations of the liver, representing additional lobes or smaller structures connected to the main liver mass. Beaver tail liver is a rare anatomic variation where the left lobe of the liver encroaches to enclose the spleen. These variants, often found by chance in patients, can create challenges in accurately distinguishing between the liver and spleen in imaging, potentially leading to misdiagnosis as splenic trauma or a subcapsular hematoma. While conducting routine dissections of the abdomen region, a variation in the size, position, and anatomical connections of the liver was noticed in a female cadaver of age 45 years. The left lobe of the liver was elongated more towards the left lateral side with some angulated narrowing after extending across the midline, encroaching the left upper quadrant of the abdomen, reaching in between the stomach and the visceral surface of the spleen, above the hilum of the spleen. The narrow end of the left lobe of the liver, placed in between the stomach and spleen, is named the hiding beaver tail liver. This variation differs from the typical beaver tail liver as well as the "kissing sign" of the liver and spleen. Unfamiliarity with such an anomaly of the liver may lead radiologists and clinicians to identify a normal anatomical variant as a pathological condition mistakenly or could confuse radiologists with fluid collections that often suggest trauma, potentially leading to fatal outcomes during invasive abdominal procedures.

## Introduction

Anatomical variations in liver lobes are infrequent and can frequently pose clinical challenges. Ectopic liver lobes, independent and in anatomical continuation with the main organ, have been reported in various locations [1,2]. Accessory liver lobes are indeed morphological variations of the liver, representing additional lobes or smaller structures connected to the main liver mass. Excessive development of accessory liver lobes could occur under the influence of several factors, including genetic predispositions, developmental abnormalities, or environmental influences. Accessory lobes are more commonly found in the right lobe of the liver, generally beneath the liver. They can have a fixed or pedunculated attachment to the main hepatic mass. Riedel's lobe is more commonly found in accessory lobes that correspond to hypertrophied segments V and VI [3]. However, it's essential to note that excessive development may lead to complications such as compression of nearby organs or interference with the function of the main liver mass. It may not pose any additional risk for hepatic pathologies [4].

The presence of a lobar appendage colloquially referred to as a "beaver tail liver" (BTL), denotes an incidental yet significant anatomical variation [3]. BTL was coined due to its resemblance to a beaver's tail in shape and appearance. BTL is a rare anatomic variation where the left lobe of the liver encroaches to enclose the spleen [2,5-7]. These variants can create challenges in accurately distinguishing between the liver and spleen in imaging, potentially leading to misdiagnosis [2,5,6]. The condition is often found by chance in patients and can be misidentified as splenic trauma or a subcapsular hematoma [2,5-8], and can create challenges in accurately distinguishing between the liver and spleen in imaging studies.

## Case presentation

**Figure 1:**
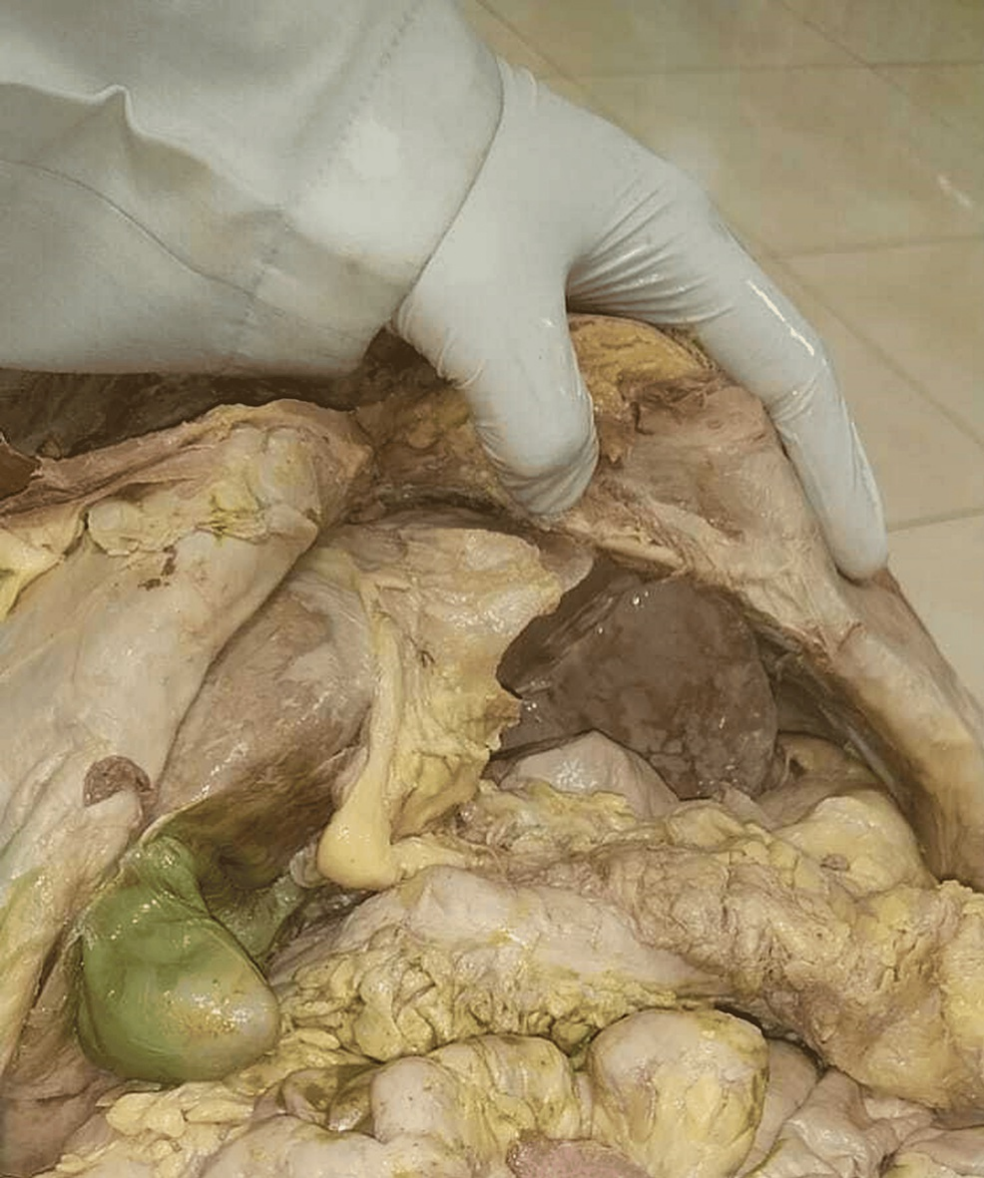
Position of the liver in the cadaver.

**Figure 2:**
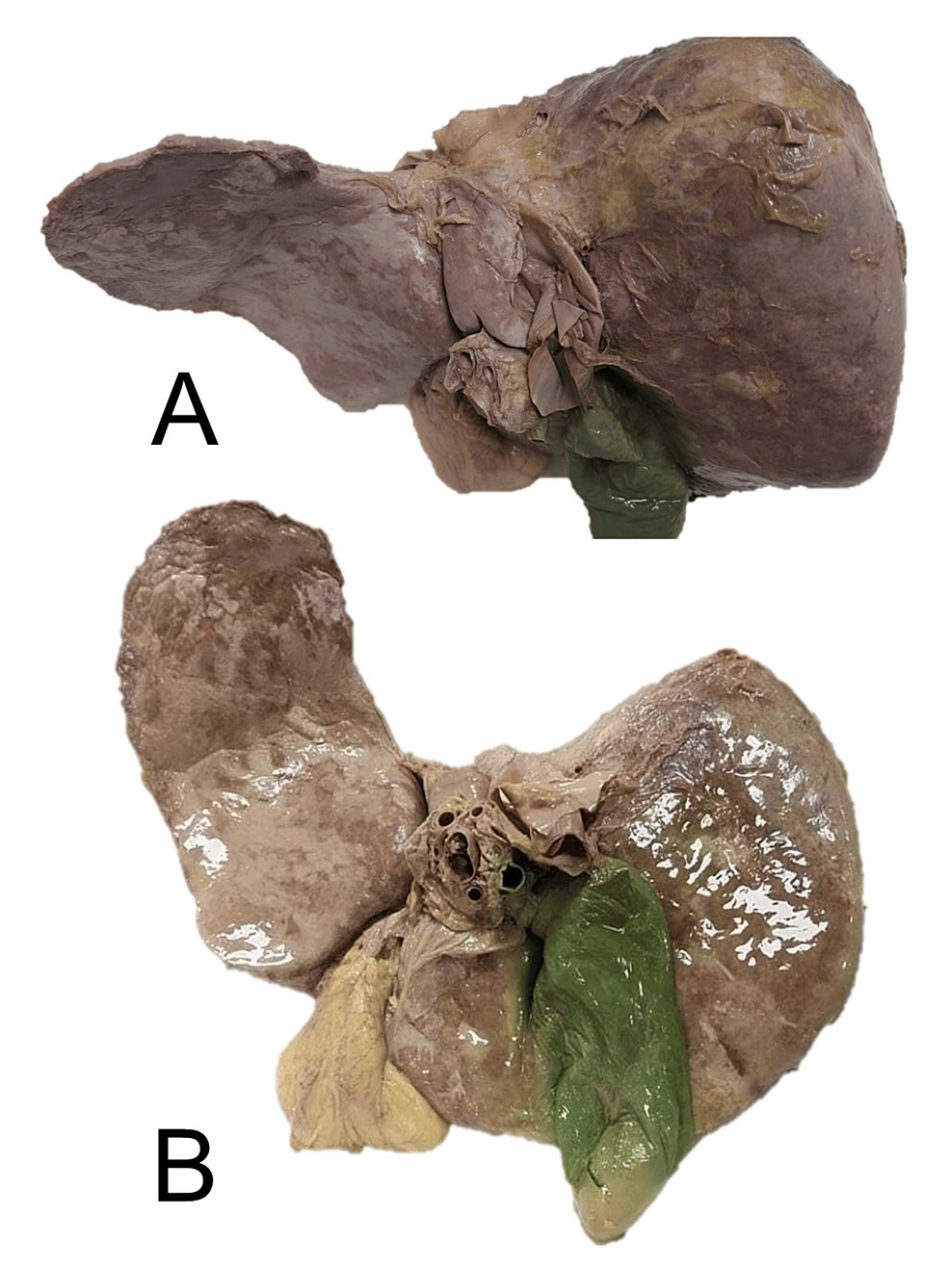
The posteroinferior aspect(A) and inferior aspect (B) of the elongated left lobe of the liver

While conducting routine cadaveric dissections of the abdomen region for undergraduate medical students, we observed discrepancies in the size, position, and anatomical connections of the liver in a female cadaver estimated to be around 45 years old. The position of the liver in the cadaver is shown in Figure 1. The left lobe of the liver appeared to be more elongated (Figure 2).

**Figure 3:**
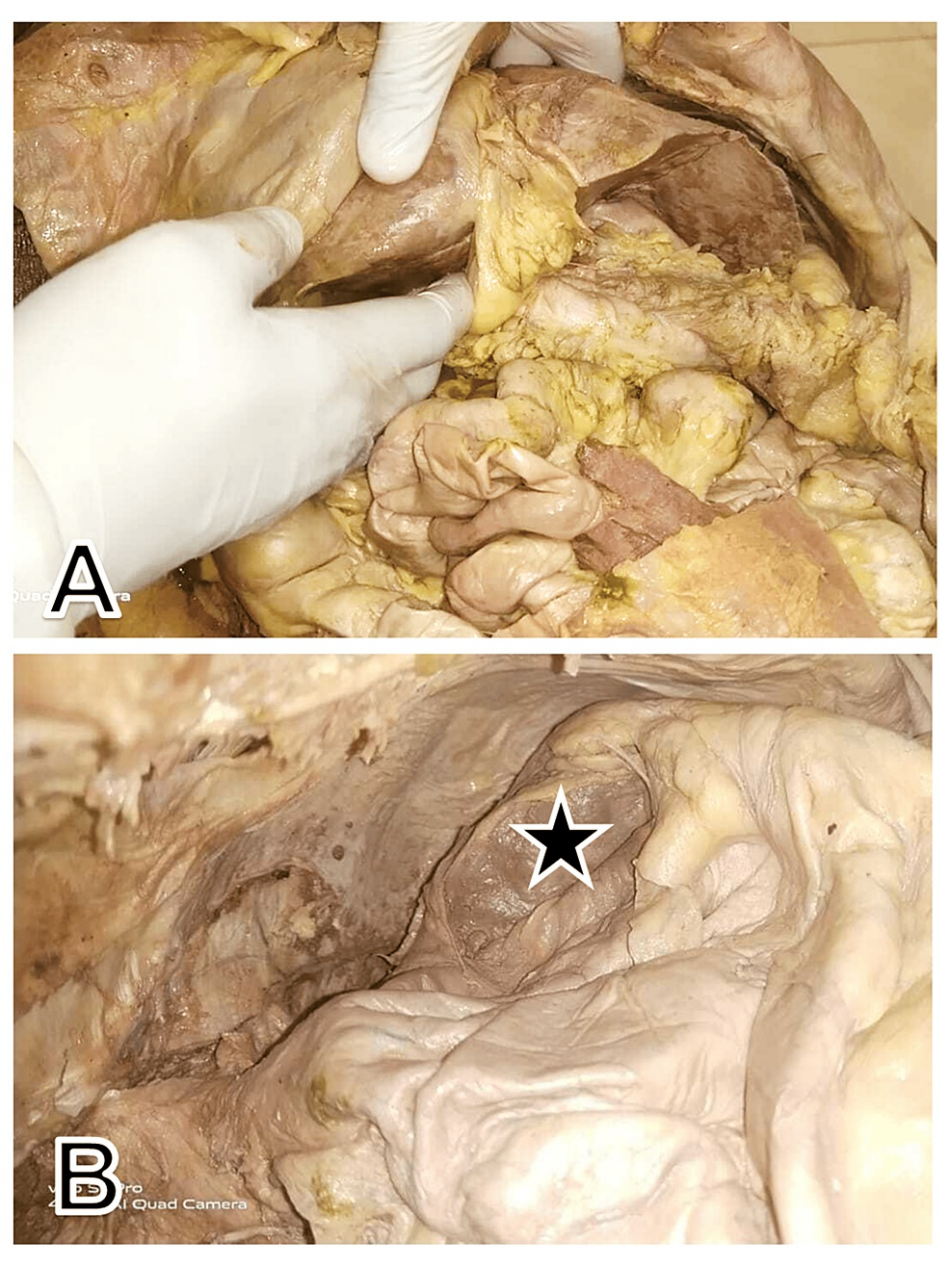
Position of the liver after removal of the anterior abdominal wall: (A) An elongated left lobe, with angulation and narrowing; (B) View of lesser sac after removal of liver and stomach. The black star denotes the visceral surface of the spleen coming in contact with the hiding beaver tail liver

The elongation of the left lobe was more towards the left lateral side, extending across the midline, encroaching on the left upper quadrant of the abdomen. An angulated narrowing of the left part of the left lobe was also observed, reaching in between the stomach and the visceral surface of the spleen, above the hilum of the spleen. After the removal of the stomach and liver during the dissection from the cadaver, a lesser sac was exposed and splenic recess could be easily visualized (Figure 3).

The spleen appeared to be of average size and dimension. The phrenicocolic ligament supported the anterior end of the spleen. The left lobe of the liver showed some adherence to the visceral surface of the spleen. Splenic blood vessels were normal in position at the hilum along with the tail of the pancreas. The fundus and body of the gall bladder were enlarged.

## Discussion

There are infrequent incidences of variations in the structure of lobes of the liver, but their presence can frequently lead to significant challenges in clinical practice. The distinctive extension of the liver's left lobe, which closely mimics the shape of a beaver's tail, has been recognized as BTL. This unique anatomical feature, notable for its lateral spread potentially encasing adjacent organs such as the spleen, contributes to its descriptive nomenclature. BTL, also called the sliver of liver, is an anatomical variant where the left lobe elongates laterally to touch and wrap around the spleen [5].

The hepatic tissue itself remains normal in this variation. When the liver and spleen exhibit similar echogenicity or density on ultrasound and CT scans, distinguishing between the two organs can be challenging. This similarity can lead to misinterpretations such as splenic trauma, perisplenic hemorrhage, or a subcapsular hematoma within the spleen [6,7]. Patients of various ages present differing clinical features to clinicians and surgeons. The presence of BTL is often identified incidentally upon investigation. This anatomical variation can influence clinical outcomes and diagnostic processes, although it may not directly correlate with the symptoms initially reported by the patient.

Sometimes, patients come with nonspecific abdominal discomfort [6], while it may also present with acute abdomen [5], or fever, haematuria, renal stone, and splenomegaly [9,10], symptoms indicative of a urinary tract infection [11]. The left hepatic lobe, when hypertrophic, adopts a distinctive shape resembling a beaver's tail. This hepatic lobe enlargement may cause a slight cranial indentation, resulting in the upward displacement of the diaphragm, which manifests as a shadow visible on chest X-rays [12]. Sometimes, it manifests as a clearly defined opaque shadow in the lower lobe of the left lung, accompanied by blunting of the costophrenic angle causing mild respiratory distress. In a case reported by Parekh et al., a CT scan of the abdomen revealed an elongated left lobe of the liver, which extended into the left hypochondrium and overlapped the spleen [13]. The fibrous appendix of the liver, also known as appendix fibrosa hepatis, is a fibrous band or a small, solid projection located on the surface of the left lobe of the liver, extending towards the diaphragm. This anatomical variation can sometimes be observed during surgical procedures or imaging studies [14]. Liver parenchyma in this variation is composed of normal tissue [7].

The "kissing sign" denotes a situation where the liver and spleen are enlarged to the extent that they touch each other. This finding is often associated with severe hepatomegaly, splenomegaly, or both, indicating an increase in the size of these organs. The recognition of the "kissing sign" can be indicative of various pathological conditions of the liver such as cirrhosis, metabolic disorders, vascular anomalies, toxicity, infections, and neoplastic growths. However, it's noteworthy that the "kissing sign" doesn't always indicate any pathological condition of the liver as it can occasionally be encountered incidentally in healthy individuals, particularly in young patients with low body fat or body mass index [15,16].

In BTL, the left liver lobe reaches the left upper quadrant with an extent exceeding the left middle axillary line and it partially encases the spleen from its anterior aspect after coming in contact with the diaphragmatic surface of the spleen [4]. In contrast, in the present study, during the dissection of a cadaver, the lateral portion of the left liver lobe was observed close to the spleen, positioned medially to its visceral surface, above the hilum of the spleen. After extending across the midline, this part of the liver lobe narrows down with an angulation, lying adjacent to the spleen. A similar liver condition was reported previously as "Hiding BTL" (HBTL) [2,17]. However, with the presence of HBTL, the liver comes in contact with the visceral surface of the spleen instead of the fundus part of the stomach. This significant anatomical relation deviation can change the expected topography within the abdominal cavity, which may impact surgical planning. Such an anomaly may lead radiologists and clinicians to mistakenly identify what is a normal anatomical variant as a pathological condition. This misinterpretation can occur especially in emergency settings where rapid decisions are made based on focused assessment with sonography in trauma and CT scans. The BTL is more susceptible to injuries resulting from trauma to the lower left chest or the upper left quadrant of the abdomen [9]. The unusual shape of the liver could be confused with fluid collections that often suggest trauma or acute disease processes [2,5,6,9,18]. Anatomical variations such as the BTL can present challenges for emergency medicine physicians, surgeons, and radiologists, mainly if they are unaware of their existence [7,12]. Unfamiliarity with this variation could potentially lead to fatal outcomes during invasive abdominal procedures [4,10,11]

## Conclusions

HBTL is a unique liver morphology where the lateral part of the left liver lobe extends across the midline and lies in close contact with the spleen's visceral surface, with an angulation. This variation differs from the typical BTL and the "kissing sign" of the liver and spleen. Medical professionals must know this hepatic variant to avoid misinterpretations in imaging studies.
